# Real-World Safety of Janus Kinase Inhibitors in Alopecia Areata: A Propensity Score-Matched Cohort Study of a Global Federated Health Network

**DOI:** 10.3390/jcm15207825

**Published:** 2026-10-10

**Authors:** Mateusz Łysek, Beata Jastrząb-Miśkiewicz, Piotr K. Krajewski

**Affiliations:** 1 Department of Dermatology, Research and Development Centre, Regional Specialist Hospital, Kamieńskiego 73a, 51-124 Wroclaw, Poland; 2 Division of Dermatology, Venereology and Clinical Immunology, Faculty of Medicine, Wroclaw University of Science and Technology, Wybrzeże Stanisława Wyspiańskiego 27, 50-370 Wroclaw, Poland; 3 Department of Dermatovenereology, 4th Military Hospital, Rudolfa Weigla 5, 50-981 Wroclaw, Poland

**Keywords:** alopecia areata, autoimmune-induced alopecia, Janus kinase inhibitors, baricitinib, ritlecitinib, tofacitinib, drug safety, real-world evidence, propensity score matching

## Abstract

**Background/Objectives:** Janus kinase inhibitors (JAKis) have transformed the treatment of alopecia areata (AA), but pivotal trials had short follow-ups and real-world long-term safety data remain scarce. We compared incident adverse outcomes between patients with AA exposed and not exposed to JAKis. **Methods:** In this retrospective cohort study of the TriNetX Global Collaborative Network, adults (≥18 years) with ≥2 diagnoses of AA (ICD-10-CM L63) and ≥2 medication events for baricitinib, tofacitinib, ritlecitinib, or oral ruxolitinib were matched 1:1 by propensity score on demographics and 13 comorbidities to patients with AA and no recorded exposure to these agents. Cox models estimated hazard ratios (HRs); *p* values for the 19 estimable outcomes were adjusted using the Benjamini–Hochberg procedure (false discovery rate 5%), and E-values assessed unmeasured confounding. Drug-specific and eight sensitivity analyses were performed. **Results:** Matching yielded 1771–1805 patients per cohort, depending on the outcome domain. Acne was more frequent with JAKis (HR, 2.425; 95% CI, 1.838–3.200; q < 0.001) in all analyses, including versus conventional immunosuppressants (HR, 1.688; 95% CI, 1.321–2.157), whereas upper respiratory tract infections (HR, 0.620; 95% CI, 0.476–0.808; q = 0.004) and urinary tract infections (HR, 0.458; 95% CI, 0.290–0.724; q = 0.006) were recorded less frequently. Cutaneous cancer was more frequent (HR, 2.639; 95% CI, 1.322–5.270; q = 0.024) and persisted after 30- and 180-day latency periods but was attenuated under a fixed 2-year follow-up and after exclusion of other JAKi indications. Severe infections, herpes zoster, and autoimmune and psychiatric outcomes did not differ significantly. **Conclusions:** In real-world AA care, JAKis were not associated with a statistically significant increase in severe infections or incident psychiatric outcomes, although power for rare serious outcomes was limited. Acne was the most consistent adverse-event signal, whereas the cutaneous cancer finding was not fully robust across specifications and requires confirmation.

## 1. Introduction

Alopecia areata (AA) is an autoimmune disease characterized by patchy, non-scarring hair loss on the scalp, face, and body, affecting approximately 2% of the global population [1,2]. Conventional therapies, including topical or intralesional corticosteroids, topical contact immunotherapies, and systemic immunosuppressants, often yield only temporary or inconsistent benefits, especially in patients with extensive or chronic disease [1,3]. The limitations of these non-specific treatments underscore the need for more effective therapeutic options for AA [1].

Emerging targeted therapies for AA have focused on the Janus kinase (JAK) pathway based on evidence that JAK–signal transducer and activator of transcription (STAT) signaling plays a central role in the autoimmune attack on hair follicles [3,4]. The introduction of JAK inhibitors (JAKis) has opened up a novel treatment avenue for AA, but it also raises important questions regarding safety [1,3]. In short-term AA trials, JAKis have generally been well tolerated; most adverse events appear to be mild to moderate and manageable, including upper respiratory tract infections, acneiform eruptions, and transient laboratory abnormalities such as hyperlipidemia [3,5]. However, clinical trials to date have been limited by short follow-up periods, leaving long-term safety outcomes insufficiently characterized [2,4].

Given their immunomodulatory properties, JAKis may carry risks of serious but rare complications with prolonged use, including opportunistic infections, thromboembolic events, and malignancies [2,6,7,8]. The lack of long-term safety data on JAKis in the context of AA constitutes a major gap in the literature [9,10]. To contribute real-world evidence toward this unmet need, we compared the incidence of adverse outcomes in patients with AA treated with JAKis versus matched comparators not exposed to JAKi therapy. By directly evaluating safety outcomes across cohorts, this study seeks to expand the evidence base on JAKi risk profiles in real-world AA populations and support data-driven decision-making in clinical practice.

## 2. Materials and Methods

### 2.1. Data Source and Study Design

We conducted a retrospective cohort study using de-identified electronic health records from the TriNetX Global Collaborative Network. This federated network aggregates de-identified electronic health record data from participating healthcare organizations across North America, Europe, Asia, and Australia. All analyses were performed on fully anonymized data in compliance with HIPAA and GDPR regulations, and thus institutional review board approval and patient consent were not required. The primary analysis was run on 2 May 2026, and the sensitivity and drug-specific analyses were run between 19 May and 27 September 2026, using data available in the TriNetX Global Collaborative Network at the time of each run. Because the network is continuously updated and cohorts are re-queried at each run, cohort sizes differ slightly between analyses; run dates and cohort sizes for each analysis are reported in Table S6. Eligible index events were restricted to the 20 years preceding each query date.

### 2.2. Study Population and Cohort Definitions

The study population consisted of adults (≥18 years) with at least two recorded diagnoses of AA (ICD-10-CM L63). We defined two cohorts. The exposure cohort included patients with at least two recorded medication events for a JAK inhibitor, whereas the comparison cohort included patients with no recorded exposure to any of the investigated JAK inhibitors. Exposure to other JAK inhibitors in the comparator pool was assessed separately (Section 3.1). JAK inhibitors included baricitinib, tofacitinib, ritlecitinib, and oral ruxolitinib. The index date was defined separately for each cohort as the date on which the patient first met all cohort-specific index-event criteria, that is, the second JAKi medication event in the exposure cohort and the second AA diagnosis in the comparison cohort; the consequences of this definition were examined in sensitivity analyses S6 and S7 (Section 2.6). Patients with missing key demographic information or invalid index dates were excluded from the cohorts.

### 2.3. Propensity Score Matching

To control for baseline differences, we performed 1:1 propensity score matching (PSM) between the JAKi and non-JAKi cohorts. Propensity scores were estimated using age at index, sex, race and ethnicity, and the following baseline characteristics: atopic dermatitis (L20), other hypothyroidism (E03), other nontoxic goiter (E04), overweight and obesity (E66), type 2 diabetes mellitus (E11), disorders of lipoprotein metabolism and other lipidemias (E78), hypertensive diseases (I10–I15), chronic kidney disease (N18), depressive episode (F32), recurrent major depressive disorder (F33), tobacco use (Z72.0), nicotine dependence (F17), and any neoplasm (C00–D49). Adequate covariate balance after matching was defined as a standardized mean difference below 0.1. Detailed characteristics before and after matching are presented in Table S1 and Figure S1. Propensity score matching was performed separately for the respective outcome-domain analyses; consequently, analytic sample sizes varied slightly across outcomes. Other approved indications for JAK inhibitors (rheumatoid arthritis, inflammatory bowel disease, psoriatic arthritis, and ankylosing spondylitis) and glucocorticoid exposure were not included in the primary propensity score model; the distribution of these conditions is reported in Table S1. Both were addressed in sensitivity analysis S8, in which patients with these conditions were excluded and glucocorticoid exposure was added to the propensity score (Table S4).

### 2.4. Drug-Specific Analyses

In addition to the pooled JAKi analysis, we conducted drug-specific safety analyses for individual JAK inhibitors (baricitinib, tofacitinib, ritlecitinib, and oral ruxolitinib). For each drug, we compared AA patients exposed to that agent with AA patients without recorded exposure to any of the four investigated JAK inhibitors using the same inclusion criteria and outcome definitions as in the primary analysis. Each drug-specific comparison was performed as an independent 1:1 PSM analysis using the same covariates and matching specifications, and HRs were estimated using Cox proportional hazards models. The drug-specific cohorts were not mutually exclusive: patients with at least two medication events for more than one agent contributed to each corresponding cohort. All drug-specific analyses were run on 26 September 2026.

### 2.5. Outcome Definitions

We evaluated incident safety outcomes across four primary groups: general adverse events (including herpes zoster (B02), severe infections requiring hospitalization (A41, J12–J18), upper respiratory tract infections (J00–J06), urinary tract infection (N39.0), and acne (L70)); autoimmune comorbidities (including rheumatoid arthritis (M05–M06), systemic lupus erythematosus (M32), psoriasis (L40), vitiligo (L80), autoimmune thyroiditis (E06.3), Sjögren syndrome (M35.0), and other immune-mediated conditions included in the composite autoimmune outcome (L80, L43, L40, L40.5, M07, E06.3, E05.0, E10, M32, M05, M06, M35.0, K50, K51, K90.0, G35)); psychiatric outcomes (including an overall psychiatric composite in the general analysis and separate subanalyses of depressive disorders (F32–F33), anxiety disorders (F40–F41), substance use disorders (F10–F19), and suicide/self-harm (T14.91, R45.851, X71–X83)); and cardiovascular complications, including venous thromboembolism (VTE; I80, I26) and a prespecified non-fatal cardiovascular composite consisting of myocardial infarction (I21) and ischemic stroke (I63). All-cause mortality was also assessed as a primary outcome. Secondary outcomes included incident hyperlipidemia (E78) and cutaneous cancer, defined as malignant melanoma of skin (C43) or other and unspecified malignant neoplasm of skin (C44); non-melanoma skin cancer (C44) was additionally analyzed separately in the drug-specific analyses. Injuries to the shoulder and upper arm (S40–S49) were assessed as a negative control outcome. To assess dermatologic surveillance bias, melanocytic nevi (D22), seborrheic keratosis (L82), and encounters for screening for malignant neoplasm of skin (Z12.83) were assessed as additional dermatologic negative control outcomes. For each outcome, patients with a prior history of the diagnosis were excluded to ensure that only incident events were captured during follow-up.

### 2.6. Sensitivity and Robustness Analyses

In addition to the pooled JAKi analysis, we conducted two prespecified sensitivity analyses: a 30-day post-index latency analysis (S1) and an analysis in which the baseline characteristics used for propensity score matching were ascertained within the 1825 days (5 years) preceding the index event rather than over the entire available record (S2). Two additional post hoc robustness analyses included a fixed 2-year follow-up window (S3) and an active-comparator analysis versus conventional systemic immunosuppressants (S4). For S4, the comparator included patients with AA receiving sulfasalazine, mycophenolic acid/mycophenolate mofetil, azathioprine, oral cyclosporine, or methotrexate and no recorded JAKi exposure. Four further post hoc analyses were performed: a 180-day post-index latency analysis to reduce the influence of diagnoses recorded shortly after treatment initiation (S5); restriction of both cohorts to cohort-qualifying events recorded on or after 1 June 2022, the month in which the first JAK inhibitor was approved for AA, so that both cohorts were drawn from the same calendar period (S6); an exposure definition requiring at least one rather than two JAKi medication events, with the index date at the first medication event (S7); and exclusion from both cohorts of patients with rheumatoid arthritis (M05–M06), inflammatory bowel disease (K50–K51), psoriatic or enteropathic arthropathy (L40.5, M07), or ankylosing spondylitis (M45), with glucocorticoid exposure (Veterans Affairs drug class HS051) added to the propensity score covariates (S8). Propensity score matching was repeated separately for each analysis using the same covariate framework as in the primary analysis, except as stated for S2 and S8.

### 2.7. Follow-Up

Outcome observation began 1 day after the index event. Patients were followed until the occurrence of the outcome or were censored at the date of the last recorded fact in their electronic health record. No fixed maximum follow-up period was imposed in the primary analysis or in S1, S2, and S4–S8; S3 restricted follow-up to days 1–730 after the index event. In S1 and S5, outcome observation began 30 and 180 days after the index event, respectively. Exposure status was defined at the index event and was not updated during follow-up.

### 2.8. Statistical Analysis

We used time-to-event analyses as the primary method to compare outcome incidence between JAKi users and non-users. Hazard ratios (HRs) with 95% confidence intervals were estimated using Cox proportional hazards models. Kaplan–Meier survival curves and log-rank tests were used for visualization and unadjusted comparisons. The proportional hazards assumption was assessed using the platform’s built-in proportionality test. To account for multiple comparisons in the pooled primary analysis, *p* values for the 19 estimable clinical outcomes were adjusted using the Benjamini–Hochberg procedure to control the false discovery rate at 5%. An FDR-adjusted q value <0.05 was considered statistically significant; other analyses are reported with nominal *p* values. E-values were calculated for associations that remained statistically significant after FDR correction to assess robustness to potential unmeasured confounding. The E-value is the minimum strength of association, on the risk ratio scale, that an unmeasured confounder would need to have with both the exposure and the outcome, conditional on the measured covariates, to fully explain away an observed association; it was calculated for the point estimate and for the confidence interval limit closest to the null [11]. Larger E-values indicate that stronger unmeasured confounding would be required to explain the association. Because the platform does not report outcome-specific person-time, approximate incidence rates per 100 person-years were derived for the fixed 2-year analysis (S3) by dividing the number of events by the product of the number of patients at risk and the cohort mean follow-up (Table S5). When fewer than 10 events occurred in a cohort, the platform suppressed the results and released no estimate; such outcomes are reported as having fewer than 10 events rather than as null findings. Cohort construction, propensity score matching, and time-to-event analyses were performed within the TriNetX Analytics platform (TriNetX, LLC, Cambridge, MA, USA). Benjamini–Hochberg-adjusted *p* values and E-values were calculated externally using IBM SPSS Statistics, version 27 (IBM Corp., Armonk, NY, USA).

## 3. Results

### 3.1. Cohort Characteristics

A total of 1871 adults with AA and at least two recorded JAKi medication events were identified, and 51,930 adults with AA and no recorded JAKi exposure formed the initial comparator pool. In the general safety analysis, PSM yielded 1799 patients in each cohort with adequate baseline balance. Detailed cohort characteristics before and after matching are presented in Table S1, and covariate balance is illustrated in Figure S1. Mean follow-up after matching was 851.1 ± 757.4 days (median, 675 days) in the JAKi cohort and 1638.1 ± 1535.8 days (median, 1185 days) in the non-JAKi cohort. Matched cohort sizes in the primary analysis ranged from 1771 to 1805 per arm across outcome domains; run dates and cohort sizes for all analyses, including the reason why cohort sizes in later runs exceed the 1871 patients identified by the original query, are given in Table S6. Of the 3315 adults with AA and at least one recorded JAKi medication event on 26 September 2026, 32.3% had only one event and were therefore not included under the primary exposure definition. In the comparator pool, 0.36% of patients had recorded exposure to other JAK inhibitors (upadacitinib, abrocitinib, or deuruxolitinib). Conditions not included in the propensity score model, including rheumatoid arthritis, were more common in the JAKi cohort after matching (Table S1).

### 3.2. General Safety Outcomes

In the PSM cohorts, JAKi exposure was not associated with a significantly different hazard of the overall general adverse events (HR, 1.111; 95% CI, 0.912–1.353; *p* = 0.296) or severe infections requiring hospitalization (HR, 1.101; 95% CI, 0.694–1.745; *p* = 0.683). The hazard of upper respiratory tract infections (HR, 0.620; 95% CI, 0.476–0.808; *p* < 0.001) and urinary tract infections (HR, 0.458; 95% CI, 0.290–0.724; *p* = 0.001) was lower in the JAKi cohort. Herpes zoster was not significantly increased in the primary analysis (HR, 1.458; 95% CI, 0.826–2.576; *p* = 0.191). Acne was more common among JAKi-treated patients (HR, 2.425; 95% CI, 1.838–3.200; *p* < 0.001). After Benjamini–Hochberg correction for multiple comparisons, the associations with acne (q < 0.001), upper respiratory tract infections (q = 0.004), and urinary tract infections (q = 0.006) remained statistically significant. Full nominal and adjusted *p* values are presented in Table S2.

Evidence against the proportional hazards assumption was observed for five outcomes in the primary analysis: urinary tract infections (PH test *p* = 0.037), anxiety (*p* = 0.002), substance use disorders (*p* = 0.010), the overall psychiatric composite (*p* = 0.010), and the composite autoimmune outcome (*p* = 0.036). The corresponding Cox HRs should therefore be interpreted as summary estimates over follow-up rather than as constant relative hazards (Table S2).

**Figure 1:**
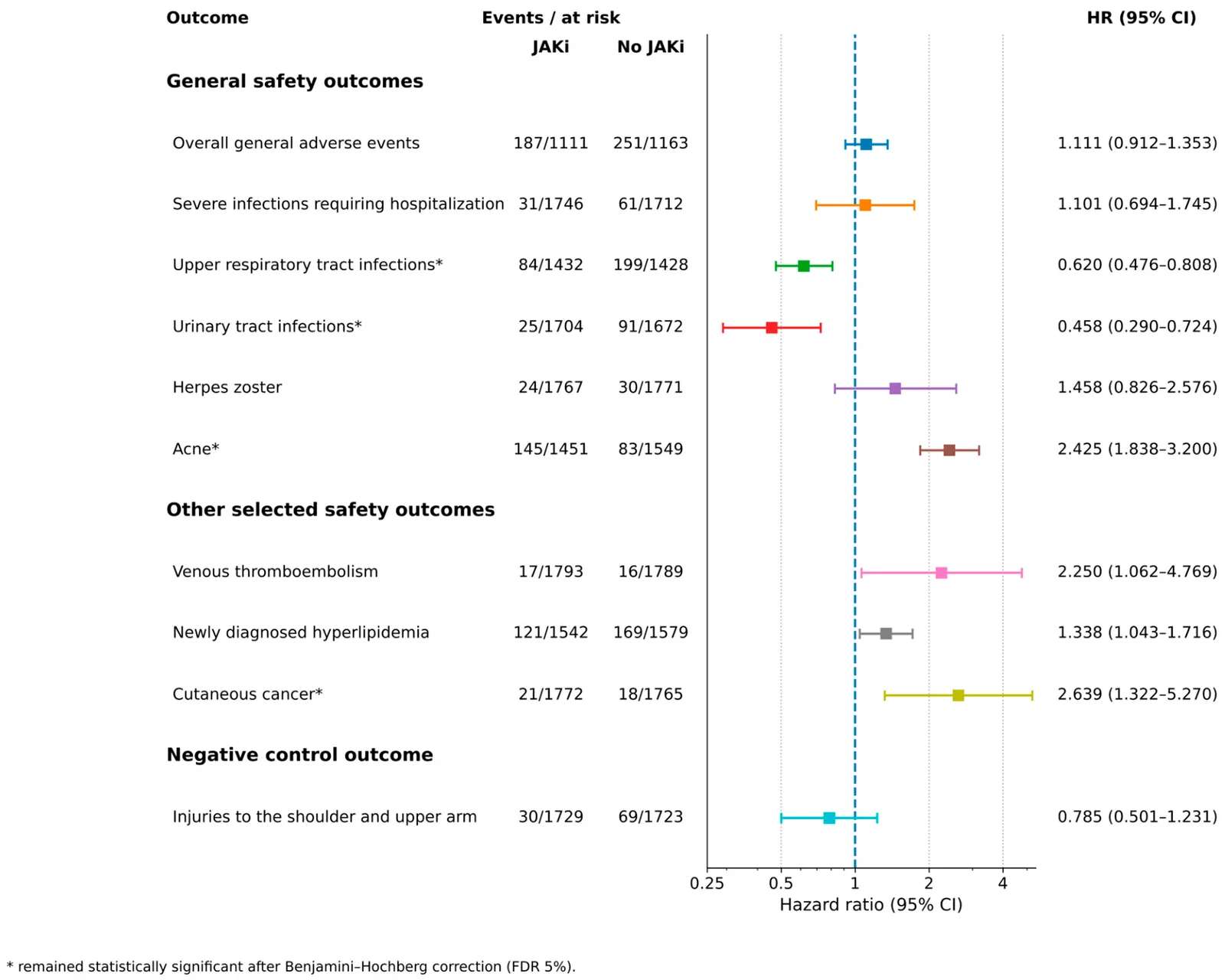
Forest plot of hazard ratios (HRs) with 95% confidence intervals for selected safety outcomes in patients with alopecia areata treated with JAK inhibitors versus propensity score-matched non-JAKi controls. Numbers shown for each outcome represent incident events/patients at risk in the JAKi and non-JAKi cohorts. Squares represent HRs, and horizontal lines represent 95% confidence intervals. The vertical dashed line represents the null value (HR = 1). Estimates are displayed on a logarithmic scale. The negative control outcome was injuries to the shoulder and upper arm. Asterisks indicate associations that remained statistically significant after Benjamini–Hochberg correction to control the false discovery rate at 5%. The negative control outcome was not included in the multiplicity correction. Abbreviations: CI, confidence interval; FDR, false discovery rate; HR, hazard ratio; JAKi, Janus kinase inhibitor.

E-values for associations that remained statistically significant after FDR correction were 2.61 for upper respiratory tract infections, 3.79 for urinary tract infections, and 4.28 for acne; the corresponding E-values for the confidence interval limits closest to the null were 1.78, 2.11, and 3.08, respectively. HRs for selected safety outcomes are summarized in Figure 1.

### 3.3. Autoimmune Outcomes

In the PSM analysis, JAKi exposure was not associated with a significantly different hazard of the composite autoimmune outcome (HR, 0.918; 95% CI, 0.647–1.302; *p* = 0.630). Individual analyses of rheumatoid arthritis, psoriasis, autoimmune thyroiditis, and Sjögren syndrome did not show statistically significant differences. For vitiligo and systemic lupus erythematosus, fewer than 10 events were recorded and the platform released no estimate; these outcomes are therefore not reported separately.

### 3.4. Psychiatric Outcomes

**Figure 2:**
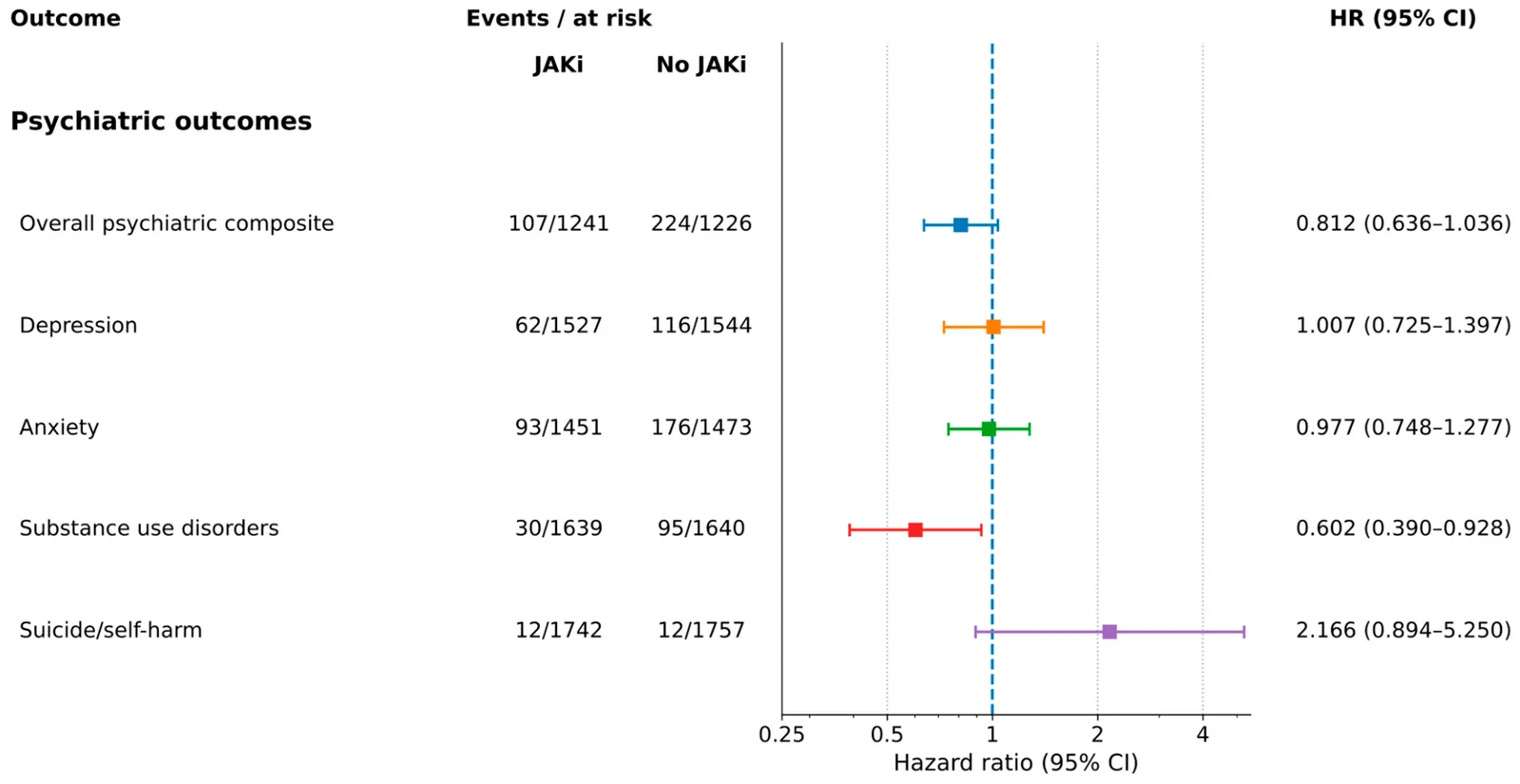
Forest plot of hazard ratios (HRs) with 95% confidence intervals for incident psychiatric outcomes in patients with alopecia areata treated with JAK inhibitors versus propensity score-matched non-JAKi controls. Numbers shown for each outcome represent incident events/patients at risk in the JAKi and non-JAKi cohorts. Squares represent HRs, and horizontal lines represent 95% confidence intervals. The vertical dashed line represents the null value (HR = 1). Estimates are displayed on a logarithmic scale. None of the psychiatric outcomes remained statistically significant after Benjamini–Hochberg correction to control the false discovery rate at 5%. Abbreviations: CI, confidence interval; HR, hazard ratio; JAKi, Janus kinase inhibitor.

The overall psychiatric composite was numerically lower in the JAKi cohort but did not reach statistical significance in the primary analysis (HR, 0.812; 95% CI, 0.636–1.036; *p* = 0.093). No significant differences were observed for depression or anxiety. Substance use disorders showed a nominally lower hazard among JAKi-treated patients (HR, 0.602; 95% CI, 0.390–0.928; *p* = 0.020), but this association did not remain statistically significant after FDR correction (q = 0.070). Suicide/self-harm was not significantly different between cohorts (HR, 2.166; 95% CI, 0.894–5.250; *p* = 0.081). Hazard ratios for incident psychiatric outcomes are summarized in Figure 2.

### 3.5. Cardiovascular Outcomes

A nominally higher hazard of venous thromboembolism was observed in the JAKi cohort (HR, 2.250; 95% CI, 1.062–4.769; *p* = 0.031), but this association did not remain statistically significant after FDR correction (q = 0.084). For the prespecified non-fatal cardiovascular composite consisting of myocardial infarction and ischemic stroke and for all-cause mortality, fewer than 10 events were recorded and no estimate was released by the platform.

### 3.6. Hyperlipidemia and Cutaneous Neoplastic Outcomes

Newly diagnosed hyperlipidemia was nominally more frequent in the JAKi cohort (HR, 1.338; 95% CI, 1.043–1.716; *p* = 0.022), but the association did not remain statistically significant after FDR correction (q = 0.070). Cutaneous cancer was more frequently recorded among JAKi-treated patients (HR, 2.639; 95% CI, 1.322–5.270; *p* = 0.005) and remained statistically significant after FDR correction (q = 0.024), although the absolute number of events was low. For cutaneous cancer, the E-value was 4.72 for the point estimate and 1.97 for the confidence interval limit closest to the null.

### 3.7. Negative Control Outcome

The negative control outcome did not differ significantly between cohorts (HR, 0.785; 95% CI, 0.501–1.231; *p* = 0.291). The dermatologic negative control outcomes also did not differ significantly: melanocytic nevi (HR, 1.197; 95% CI, 0.893–1.604; *p* = 0.229), seborrheic keratosis (HR, 1.279; 95% CI, 0.950–1.724; *p* = 0.104), and encounters for skin cancer screening (HR, 1.153; 95% CI, 0.695–1.912; *p* = 0.580); event counts and the run date are given in Table S6.

### 3.8. Drug-Specific Safety Analyses

Drug-specific analyses are summarized in Table 1. Acne was more common and upper respiratory tract infections were less common with baricitinib and tofacitinib; for ritlecitinib, the estimate for acne was not statistically significant, and fewer than 10 upper respiratory tract infection events were recorded. Baricitinib was also associated with higher hazards of hyperlipidemia and cutaneous cancer. No drug-specific differences were observed for psychiatric outcomes. For oral ruxolitinib (42 matched patients per arm), fewer than 10 events were recorded for every outcome, and no estimate could be obtained.

### 3.9. Results of Sensitivity and Robustness Analyses

Sensitivity analyses S1, S2, S3, and S5 varied the analytic window (Table S3), and S4 and S6–S8 varied the study population or the comparator (Table S4). Acne was more common among JAKi-treated patients in all eight sensitivity analyses, and upper respiratory tract and urinary tract infections were less common in all eight; severe infections were not significantly increased in any analysis. Herpes zoster showed a nominally higher hazard only in S2, with evidence against the proportional hazards assumption. The psychiatric composite did not differ significantly in S1 or S2; nominally lower hazards were observed for substance use disorders only in S2 and S6 and for the psychiatric composite only in S6 and S8. VTE was nominally elevated in S1 but not in S2 or S7, and fewer than 10 events were recorded in the remaining analyses. Cutaneous cancer remained elevated in both S1 and S2. In S1 and S2, fewer than 10 events were recorded for suicide/self-harm and Sjögren syndrome.

In the fixed 2-year follow-up analysis (S3), propensity score matching yielded 2161 patients in each cohort. The higher hazard of acne and lower hazards of upper respiratory tract and urinary tract infections persisted, while severe infections remained non-significantly increased. Hyperlipidemia remained elevated, whereas the association with cutaneous cancer was attenuated and no longer statistically significant. Neither the psychiatric composite nor substance use disorders differed significantly between cohorts. All five proportional-hazards violations identified in the primary analysis were no longer present after restriction to the 2-year follow-up window; however, evidence against the proportional-hazards assumption was observed for the overall general adverse-event composite in S3 (Table S3). Approximate incidence rates per 100 person-years in S3 are given in Table S5.

In the general-safety active-comparator analysis (S4), propensity score matching yielded 1805 patients in each cohort. Acne remained more frequent among JAKi-treated patients, although the association was attenuated and hyperlipidemia was also more frequently recorded. Upper respiratory tract and urinary tract infections remained less common, and a lower overall hazard of severe infections was observed compared with conventional systemic immunosuppressants. However, the proportional-hazards assumption was violated for urinary tract and severe infections. The psychiatric composite and substance use disorders were not significantly different between groups, whereas cutaneous cancer remained nominally elevated. Autoimmune outcomes were not interpreted because of the potential for confounding by indication in the active-comparator cohort (Table S4).

In the additional post hoc four sensitivity analyses (S5–S8), acne remained more common and upper respiratory and urinary tract infections less common among JAKi-treated patients. Cutaneous cancer remained more common in S5 and S7 but was no longer statistically significant in S8, and fewer than 10 events were recorded in S6. Hyperlipidemia was more common in S5 and S7 but not in S6 or S8. Restricting both cohorts to the period from June 2022 (S6) reduced the imbalance in follow-up between cohorts. The injury negative control outcome did not differ significantly in any analysis, although point estimates were below 1 in most (Table S3 and Table S4).

## 4. Discussion

In this large, real-world, propensity score-matched cohort of patients with AA, the primary analysis identified higher hazards of acne and cutaneous cancer and lower recorded hazards of upper respiratory tract and urinary tract infections after FDR correction. Across eight sensitivity analyses, the associations with acne and common infections were consistent, including after restriction of follow-up to 2 years, use of an active immunosuppressant comparator, restriction to the post-approval period, indexing at the first medication event, and exclusion of other JAKi indications. The cutaneous cancer association persisted after latency periods of 30 and 180 days but was attenuated with a fixed 2-year follow-up and after exclusion of other JAKi indications, and hyperlipidemia was inconsistent across specifications. No increased hazard of severe infections was identified in any analysis. These findings highlight the importance of follow-up duration, calendar period, and comparator selection when interpreting real-world JAKi safety data [12].

Herpes zoster was not significantly increased in the primary analysis or in the other sensitivity analyses in which it could be estimated, although a nominally significant difference emerged in the second sensitivity analysis, with evidence against the proportional hazards assumption. The inconsistency across analytic specifications, together with the small number of incident events, limits the robustness of this signal. From a mechanistic perspective, cytokines involved in antiviral immune surveillance signal, including IFN-γ and IL-15, through the JAK–STAT pathway, and their inhibition may impair host defences against latent viral infections. This provides biological plausibility for the increased incidence of herpes zoster previously reported in randomized trials and postmarketing data [12,13,14,15,16]. In the fixed 2-year analysis, the approximate incidence of herpes zoster among JAKi-treated patients was 0.44 per 100 person-years (Table S5), lower than the 0.9 per 100 patient-years reported for ritlecitinib in the integrated ALLEGRO program [17] and the 1.9 per 100 patient-years reported for baricitinib in the long-term BRAVE-AA data [18]; this difference most likely reflects under-recording of herpes zoster in routine health records rather than a lower risk. Nevertheless, the absence of an increased hazard in the fixed 2-year, active-comparator and other sensitivity analyses further indicates that our data do not establish a consistent increase in herpes zoster risk among patients with AA treated with JAKis.

The higher hazard of acne was the most consistent adverse-event signal in our study, which remained statistically significant after FDR correction in the primary analysis and persisted in all eight sensitivity analyses. This observation is consistent with previous evidence: acne was reported more frequently with baricitinib than with placebo in the BRAVE-AA trials [19], acne was also reported with ritlecitinib in the ALLEGRO phase 2b/3 trial [20], and acneiform and folliculitis-like eruptions have been among the most common adverse events in real-world studies of baricitinib and ritlecitinib, with an incidence ranging from approximately 6% to 21% [21,22]. Because patients receiving JAKis are likely to be followed more closely by dermatologists, detection bias may have inflated recorded acne; however, the association persisted after 30- and 180-day latency periods and in the active-comparator analysis, and the dermatologic negative control outcomes did not differ significantly between cohorts, which argues against surveillance alone as the explanation.

In contrast, the lower recorded hazards of upper respiratory tract and urinary tract infections were unexpected, particularly because upper respiratory tract infections have been commonly reported during JAKi treatment in clinical trials and pharmacovigilance studies [12,15]. Both associations remained statistically significant after Benjamini–Hochberg correction in the primary analysis. They were also consistent in all eight sensitivity analyses. The disappearance of the proportional-hazards violation for urinary tract infections after restricting follow-up suggests that unequal or extended follow-up may have contributed to the primary finding. Persistence of lower infection hazards versus conventional systemic immunosuppressants also makes differences in healthcare utilization alone a less likely explanation and may partly reflect a higher infection burden associated with conventional immunosuppression rather than a protective effect of JAKis. However, point estimates for the injury negative control outcome were also below 1 in most analyses (range, 0.622–1.045), suggesting that part of the lower recorded infection hazard may reflect less frequent contact with healthcare for acute conditions. Residual confounding, diagnostic coding, and treatment selection remain possible. Severe infections were not increased in the primary analysis or in any sensitivity analysis; although a lower hazard was observed in S4, the proportional-hazards assumption was violated, precluding interpretation as a constant protective effect.

No consistent association between JAKi exposure and incident psychiatric outcomes was identified. The overall psychiatric composite was not significantly different in the primary analysis or in six of eight sensitivity analyses; hazard ratios were below 1 in all analyses and reached nominal significance only after restriction to the post-approval period (S6) and after exclusion of other JAKi indications (S8). The nominally lower hazard of substance use disorders observed in the primary analysis was not reproduced consistently. Depression and anxiety likewise showed no consistent between-group differences. This is relevant given the substantial psychosocial burden of AA [23,24], reports of improved psychological outcomes accompanying clinical response to baricitinib [25], and emerging evidence linking JAKi treatment with changes in systemic inflammatory markers, as inflammation has also been associated with depressive symptoms [26,27]. However, our findings do not establish a psychiatric effect of JAK inhibition, and the direction of the estimates may reflect residual confounding or differences in healthcare contact.

Concerns regarding cardiovascular and thromboembolic safety of JAKis have arisen mainly from rheumatology populations with higher baseline cardiovascular risk [28,29]. In contrast, a meta-analysis of randomized trials in immune-mediated skin diseases found no significant increase in MACE or VTE with short-term JAKi exposure [30]. In our study, a nominally higher hazard of VTE was observed in the primary analysis, but the association did not remain statistically significant after FDR correction. VTE was nominally elevated in the 30-day latency analysis but not in S2 or when exposure was defined by a single medication event (S7), and fewer than 10 events were recorded in the remaining sensitivity analyses, underscoring the instability of this rare-event estimate. The primary finding should therefore be considered exploratory rather than evidence of a causal association. For the non-fatal cardiovascular composite and all-cause mortality, fewer than 10 events were recorded and no estimate was available.

JAKi exposure was not associated with the composite autoimmune outcome. The cutaneous cancer association was more persistent than a chance finding would suggest: it remained after latency periods of 30 and 180 days, which reduces the likelihood that it reflects diagnoses prompted by examination at treatment initiation, and it was not accompanied by significant differences in dermatologic negative control outcomes (melanocytic nevi, seborrheic keratosis, and skin cancer screening encounters; HRs, 1.15–1.28, with upper confidence limits of 1.60–1.91), suggesting that increased dermatologic surveillance alone does not explain it, although a modest surveillance effect cannot be excluded. It was also observed for baricitinib in the drug-specific analyses. However, the association was attenuated and no longer statistically significant with a fixed 2-year follow-up and after exclusion of patients with other JAKi indications, event counts were low, the proportional hazards assumption was violated in two sensitivity analyses, and the E-value for the confidence interval limit closest to the null was 1.97. The role of JAK–STAT signaling in cancer biology is context-dependent [31]. A higher incidence of non-melanoma skin cancer has been reported with tofacitinib than with tumor necrosis factor inhibitors in patients with rheumatoid arthritis [29], and a meta-analysis across indications found a higher incidence of malignancy, including non-melanoma skin cancer, with JAKis than with tumor necrosis factor inhibitors but not with placebo or methotrexate [32]. Conversely, the incidence of non-melanoma skin cancer in the long-term BRAVE-AA data was low [18], and no short-term malignancy signal was found in a multicenter AA cohort [33]. Given the low number of events and the absence of data on ultraviolet exposure and skin phototype, these findings should be considered hypothesis-generating and require confirmation in larger cohorts with longer and more uniformly ascertained follow-up.

Newly diagnosed hyperlipidemia did not meet the FDR threshold in the primary analysis and was inconsistent across specifications: it was more frequently recorded in the 30- and 180-day latency, fixed 2-year, active-comparator, and single-medication-event analyses but not in S2, after restriction to the post-approval period (S6), or after exclusion of other JAKi indications (S8). Increases in low- and high-density lipoprotein cholesterol were observed with baricitinib in the BRAVE-AA trials [19], and effects of JAK inhibition on serum lipid levels have been reported previously [34]. However, our outcome was based on diagnostic coding rather than laboratory measurements and may have underascertained treatment-emergent lipid abnormalities that were not recorded as clinical diagnoses.

This retrospective EHR-based study is subject to residual confounding and selection bias despite PSM, particularly because disease severity measures such as Severity of Alopecia Tool (SALT) scores, disease duration, prior treatment lines, and AA-related visit counts were unavailable and may have influenced treatment selection; residual confounding by severity may have contributed to positive associations such as those for acne and cutaneous cancer. Glucocorticoid exposure, a partial marker of disease severity, was included in the propensity score only in S8, and other JAKi indications were more frequent in the exposure cohort after primary matching (Table S1). Requiring two medication events excluded 32.3% of JAKi-exposed patients with a single recorded event, some of whom may have discontinued treatment early because of adverse events, and created a period of immortal time between the first and second events; the analysis indexed at the first medication event (S7) yielded directionally similar findings. Exposure was fixed at the index date, and dose and cumulative treatment duration were not analyzed. Index dates corresponded to the second AA diagnosis in comparators and to the second JAKi medication event in exposed patients; restriction to the post-approval period (S6) reduced calendar-time differences but cannot fully align disease stage at index. The comparator excluded only the four investigated agents, but only 0.36% of the comparator pool had recorded exposure to other JAK inhibitors. Outcomes were identified using diagnostic codes, introducing potential misclassification and underreporting, particularly for mild events and laboratory abnormalities not recorded as diagnoses. Available follow-up differed substantially between the primary cohorts, potentially contributing to differential outcome ascertainment and time-dependent associations. Restricting follow-up to a common 2-year window substantially reduced this imbalance, and all five proportional-hazards violations identified in the primary analysis were no longer present. Although nonproportionality was observed for the overall general adverse-event composite in S3, the disappearance of the primary violations suggests that unequal or extended follow-up may have contributed to the original pattern of nonproportionality. However, differential censoring and differences in healthcare utilization cannot be fully excluded, and point estimates below 1 for the injury negative control outcome in most analyses suggest less frequent healthcare contact in the JAKi cohort. The platform suppresses results when fewer than 10 events occur so several rare outcomes, including all outcomes for oral ruxolitinib, could not be estimated; these represent missing information rather than an absence of risk. Incidence rates were approximated from cohort-level mean follow-up because outcome-specific person-time is not provided by the platform, and the VTE definition (I80, I26) included phlebitis and thrombophlebitis and did not include other venous embolism and thrombosis (I82). The active-comparator analysis reduced concerns related to comparator selection but included a heterogeneous group of conventional systemic immunosuppressants and retained differences in available follow-up; these estimates should therefore be considered supportive rather than confirmatory. Analyses were not powered to assess dose-related safety differences among individual JAK inhibitors, and follow-up may have been insufficient to detect rare or long-term events. Finally, as this was a nonrandomized study, causality cannot be inferred, and unmeasured factors such as treatment adherence, exposure duration, and prescribing patterns may have influenced the observed associations.

## 5. Conclusions

In this real-world, propensity score-matched cohort study, JAK inhibitor therapy for alopecia areata was not associated with a statistically significant increase in severe infections, incident psychiatric outcomes, or autoimmune comorbidities, although power for rare serious outcomes was limited. The higher hazard of acne was the most consistent adverse-event association and persisted across all eight sensitivity analyses, including those using a fixed follow-up window and an active comparator. Lower recorded hazards of upper respiratory and urinary tract infections were also consistent across analytic specifications, although these findings should not be interpreted as evidence of a protective effect. The cutaneous cancer association persisted after latency periods of 30 and 180 days but was attenuated with a fixed follow-up window and after exclusion of other JAKi indications, and hyperlipidemia was inconsistent across specifications. No consistent associations with psychiatric outcomes were identified, and no increased hazard of severe infections was observed in any analysis.

Overall, these data are reassuring with respect to the safety of JAK inhibitors in alopecia areata over a follow-up of up to approximately 2 years, while indicating that the cutaneous malignancy signal requires confirmation in larger cohorts with longer and more uniformly ascertained follow-up. In clinical practice, monitoring for acneiform eruptions, periodic assessment of lipid profiles, and routine skin examination appear warranted, and follow-up duration, calendar period, and comparator selection should be considered carefully when interpreting real-world safety data on JAK inhibitors.

## Figures and Tables

**Table 1:** Drug-specific safety outcomes in patients with alopecia areata, with incident events and patients at risk.

**Outcome**	**Baricitinib**	**Tofacitinib**	**Ritlecitinib**	**Oral Ruxolitinib**
**General safety outcomes**	*matched n = 1241 per arm*	*matched n = 778 per arm*	*matched n = 485 per arm*	*matched n = 42 per arm*
Overall general adverse events	1.188 (0.913–1.546) * p = 0.200 *107/777 vs. 181/803*	0.995 (0.756–1.309) * p = 0.972 *104/469 vs. 112/489*	0.761 (0.457–1.268) *p* = 0.294 *23/288 vs. 69/298*	*<10 events* *estimate suppressed*
Herpes zoster	*<10 events* *estimate suppressed*	1.684 (0.807–3.513) *p* = 0.160 *19/762 vs. 14/767*	*<10 events* *estimate suppressed* *<10/477 vs. 12/478*	*<10 events* *estimate suppressed*
Severe infections requiring hospitalization	0.627 (0.304–1.292) *p* = 0.202 *11/1205 vs. 54/1191*	1.121 (0.610–2.060) *p* = 0.712 *22/740 vs. 25/742*	*<10 events* *estimate suppressed*	*<10 events* *estimate suppressed*
Upper respiratory tract infections	0.600 (0.415–0.868) *p* = 0.006 *43/989 vs. 134/955*	0.633 (0.448–0.896) *p* = 0.009 *56/602 vs. 84/578*	*<10 events* *estimate suppressed*	*<10 events* *estimate suppressed*
Urinary tract infections	*<10 events* *estimate suppressed*	0.610 (0.349–1.064) *p* = 0.079 *20/719 vs. 36/725*	*<10 events* *estimate suppressed*	*<10 events* *estimate suppressed*
Acne	2.780 (1.937–3.988) *p* < 0.001 *89/1004 vs. 64/1093*	1.814 (1.244–2.646) *p* = 0.002 *72/632 vs. 46/681*	1.589 (0.922–2.738) *p* = 0.093 *27/368 vs. 33/422*	*<10 events* *estimate suppressed*
**Psychiatric outcomes**	*matched n = 1242 per arm*	*matched n = 778 per arm*	*matched n = 485 per arm*	*matched n = 42 per arm*
Overall psychiatric composite	0.846 (0.589–1.217) *p* = 0.367 *49/868 vs. 135/871*	0.835 (0.609–1.145) *p* = 0.263 *70/532 vs. 104/541*	*<10 events* *estimate suppressed*	*<10 events* *estimate suppressed*
Depression	1.038 (0.637–1.691) *p* = 0.881 *29/1068 vs. 66/1086*	0.802 (0.535–1.201) *p* = 0.283 *41/668 vs. 69/701*	*<10 events* *estimate suppressed*	*<10 events* *estimate suppressed*
Anxiety	1.076 (0.721–1.606) *p* = 0.720 *44/1004 vs. 105/1018*	0.818 (0.572–1.171) *p* = 0.272 *53/634 vs. 86/654*	1.312 (0.580–2.968) *p* = 0.513 *11/387 vs. 40/411*	*<10 events* *estimate suppressed*
Substance use disorders	0.659 (0.329–1.318) *p* = 0.235 *12/1150 vs. 48/1151*	1.148 (0.653–2.019) *p* = 0.632 *25/720 vs. 32/723*	*<10 events* *estimate suppressed*	*<10 events* *estimate suppressed*
Suicide/self-harm	*<10 events* *estimate suppressed*	*<10 events* *estimate suppressed*	*<10 events* *estimate suppressed*	*<10 events* *estimate suppressed*
**Autoimmune outcomes**	*matched n = 1241 per arm*	*matched n = 778 per arm*	*matched n = 485 per arm*	*matched n = 42 per arm*
Composite autoimmune outcome	0.917 (0.575–1.462) *p* = 0.716 *30/994 vs. 75/1065*	0.831 (0.506–1.364) *p* = 0.463 *27/543 vs. 41/675*	*<10 events* *estimate suppressed*	*<10 events* *estimate suppressed*
Rheumatoid arthritis	*<10 events* *estimate suppressed*	1.324 (0.547–3.208) *p* = 0.533 *11/687 vs. 11/764*	*<10 events* *estimate suppressed*	*<10 events* *estimate suppressed*
Psoriasis	*<10 events* *estimate suppressed*	*<10 events* *estimate suppressed*	*<10 events* *estimate suppressed*	*<10 events* *estimate suppressed*
Autoimmune thyroiditis	1.392 (0.589–3.289) *p* = 0.449 *11/1179 vs. 17/1201*	1.014 (0.438–2.350) *p* = 0.974 *11/725 vs. 12/756*	*<10 events* *estimate suppressed* *<10/460 vs. 11/476*	*<10 events* *estimate suppressed*
**Other outcomes**	*matched n = 1241 per arm*	*matched n = 778 per arm*	*matched n = 485 per arm*	*matched n = 42 per arm*
Cutaneous cancer (C43 + C44)	2.381 (1.005–5.644) *p* = 0.043 *13/1217 vs. 20/1216*	*<10 events* *estimate suppressed*	*<10 events* *estimate suppressed*	*<10 events* *estimate suppressed*
Non-melanoma skin cancer (C44)	*<10 events* *estimate suppressed*	*<10 events* *estimate suppressed*	*<10 events* *estimate suppressed*	*<10 events* *estimate suppressed*
Hyperlipidemia	1.854 (1.347–2.553) *p* < 0.001 *93/1052 vs. 145/1054*	1.269 (0.883–1.825) *p* = 0.198 *64/647 vs. 69/679*	1.675 (0.796–3.523) *p* = 0.170 *16/416 vs. 33/422*	*<10 events* *estimate suppressed*
Venous thromboembolism	*<10 events* *estimate suppressed*	*<10 events* *estimate suppressed*	*<10 events* *estimate suppressed*	*<10 events* *estimate suppressed*
Negative control outcome: injuries to the shoulder and upper arm	0.670 (0.335–1.340) *p* = 0.255 *12/1183 vs. 48/1178*	0.765 (0.413–1.414) * p = 0.391 *18/732 vs. 31/751*	*<10 events* *estimate suppressed*	*<10 events* *estimate suppressed*

Each drug-specific comparison was an independent 1:1 propensity score-matched analysis of adults with at least two diagnoses of alopecia areata and at least two medication events for the drug shown, versus adults with at least two diagnoses of alopecia areata and no recorded exposure to any of the four investigated JAK inhibitors. Cohort definitions, index event, covariate set and outcome definitions were identical to the pooled primary analysis; the outcome window began 1 day after the index event with no maximum follow-up. All analyses were run on 26 September 2026. Propensity score matching was performed separately for each outcome domain; the matched cohort size per arm is therefore given for each domain. Values are hazard ratios (HRs) with 95% confidence intervals (CIs) from Cox proportional hazards models; *p* values are nominal and derived from log-rank tests. Italicised figures below each estimate are incident events/patients at risk in the exposed cohort versus the comparator cohort, after exclusion of patients with the outcome before the start of the time window. * Evidence against the proportional hazards assumption (PH test *p* < 0.05); the corresponding HR should be interpreted as a summary estimate over follow-up rather than as a constant relative hazard. The drug-specific cohorts are not mutually exclusive: a patient with at least two medication events for more than one JAK inhibitor contributes to each corresponding cohort. Before matching, the four drug cohorts comprised 1298, 778, 499 and 42 patients, a total of 2617 against 2245 in the pooled JAKi cohort queried the same day, so at least 372 patients contributed to more than one drug-specific cohort. “<10 events” indicates that the outcome was analysed but the patient count was below the reporting threshold of the TriNetX platform, which then returns “Based on the outcome terms, the patient count is too small, so detailed results cannot be displayed” and releases no counts or estimate. These cells denote absence of information, not absence of effect, and must not be read as showing no difference. Where a count is shown alongside, the platform released the comparator count only. No estimate could be obtained for any outcome in the oral ruxolitinib cohort (42 matched patients per arm). Abbreviations: CI, confidence interval; HR, hazard ratio; JAKi, Janus kinase inhibitor; PH, proportional hazards.

## Data Availability

The data underlying this study were obtained from the TriNetX Global Collaborative Network and are not publicly available because of licensing restrictions. Aggregated data supporting the reported findings are presented within the article and its Supplementary Materials. Access to the TriNetX platform may be requested from TriNetX, LLC (https://trinetx.com/), for researchers who meet the criteria for access to de-identified data.

## Supplementary Materials

The following supporting information can be downloaded at: https://www.mdpi.com/article/10.3390/jcm15207825/s1, Table S1: Baseline characteristics of the cohorts before and after propensity score matching; Table S2: Primary pooled safety outcomes with nominal and Benjamini–Hochberg-adjusted *p* values and proportional hazards testing; Table S3: Sensitivity analyses varying the analytic window, with incident events and patients at risk; Table S4: Sensitivity analyses varying the study population or the comparator, with incident events and patients at risk; Table S5: Approximate incidence rates per 100 person-years in the fixed 2-year follow-up analysis (S3); Table S6: Analysis run dates and cohort sizes; Figure S1: Love plot showing baseline covariate balance before and after propensity score matching in patients with alopecia areata treated with JAK inhibitors versus non-users.

## Abbreviations

The following abbreviations are used in this manuscript:

AA	alopecia areata
CI	confidence interval
EHR	electronic health record
FDR	false discovery rate
GDPR	General Data Protection Regulation
HIPAA	Health Insurance Portability and Accountability Act
HR	hazard ratio
ICD-10-CM	International Classification of Diseases, Tenth Revision, Clinical Modification
JAK	Janus kinase
JAKi	Janus kinase inhibitor
MACE	major adverse cardiovascular events
PH	proportional hazards
PSM	propensity score matching
SALT	Severity of Alopecia Tool
STAT	signal transducer and activator of transcription
VTE	venous thromboembolism
