# Supplementary material for: Real-World Safety of Janus Kinase Inhibitors in Alopecia Areata: A Propensity Score-Matched Cohort Study of a Global Federated Health Network
Source: J Clin Med. 2026 Oct 10;15(20):7825. doi: 10.3390/jcm15207825 (PMC13705346; doi:10.3390/jcm15207825)
Supplement: Supplementary file 1 [file jcm-15-07825-s001.zip › jcm-4579218-supplementary.pdf]

Supplementary Materials

Real-World Safety of Janus Kinase Inhibitors in Alopecia Areata: A Propensity Score-Matched Cohort Study of a Global Federated Health Network

Mateusz Łysek, Beata Jastrząb-Miśkiewicz and Piotr K. Krajewski

Contents: Table S1, Table S2, Table S3, Table S4, Table S5, Table S6, Figure S1.

Table S1. Baseline characteristics of the cohorts before and after propensity score matching.

Characteristic	AA with JAKi (before, n = 1,799)	AA without JAKi (before, PSM pool n = 48,367)	SMD (before)	AA with JAKi (after, n = 1,799)	AA without JAKi (after, n = 1,799)	SMD (after)
**Demographics**						
Age at index, years, mean ± SD	37.0 ± 16.7	38.0 ± 18.1	0.062	37.0 ± 16.7	37.0 ± 17.4	0.004
Female sex, n (%)	1,163 (64.6%)	28,650 (61.6%)	0.064	1,163 (64.6%)	1,147 (63.8%)	0.019
White race, n (%)	1,096 (60.9%)	21,407 (46.0%)	0.303	1,096 (60.9%)	1,104 (61.4%)	0.009
Black or African American race, n (%)	205 (11.4%)	6,405 (13.8%)	0.071	205 (11.4%)	200 (11.1%)	0.009
Not Hispanic or Latino ethnicity, n (%)	1,215 (67.5%)	25,433 (54.7%)	0.267	1,215 (67.5%)	1,238 (68.8%)	0.027
Hispanic or Latino ethnicity, n (%)	145 (8.1%)	5,360 (11.5%)	0.117	145 (8.1%)	137 (7.6%)	0.017
**Baseline diagnoses**						
Atopic dermatitis (L20), n (%)	234 (13.0%)	2,483 (5.3%)	0.268	234 (13.0%)	215 (12.0%)	0.032
Other hypothyroidism (E03), n (%)	188 (10.5%)	3,681 (7.9%)	0.088	188 (10.5%)	146 (8.1%)	0.081
Other nontoxic goiter (E04), n (%)	63 (3.5%)	1,661 (3.6%)	0.004	63 (3.5%)	52 (2.9%)	0.035
Overweight and obesity (E66), n (%)	210 (11.7%)	5,844 (12.6%)	0.027	210 (11.7%)	188 (10.5%)	0.039
Type 2 diabetes mellitus (E11), n (%)	73 (4.1%)	2,678 (5.8%)	0.079	73 (4.1%)	64 (3.6%)	0.026
Disorders of lipoprotein metabolism and other lipidemias (E78), n (%)	259 (14.4%)	8,033 (17.3%)	0.079	259 (14.4%)	221 (12.3%)	0.062
Hypertensive diseases (I10-I15), n (%)	201 (11.2%)	7,284 (15.7%)	0.132	201 (11.2%)	176 (9.8%)	0.045
Chronic kidney disease (N18), n (%)	39 (2.2%)	1,070 (2.3%)	0.009	39 (2.2%)	38 (2.1%)	0.004
Depressive episode (F32), n (%)	224 (12.5%)	5,222 (11.2%)	0.038	224 (12.5%)	203 (11.3%)	0.036
Major depressive disorder, recurrent (F33), n (%)	75 (4.2%)	1,670 (3.6%)	0.030	75 (4.2%)	69 (3.8%)	0.017
Tobacco use (Z72.0), n (%)	28 (1.6%)	1,039 (2.2%)	0.050	28 (1.6%)	27 (1.5%)	0.005
Nicotine dependence (F17), n (%)	82 (4.6%)	2,904 (6.2%)	0.074	82 (4.6%)	71 (3.9%)	0.030
Any neoplasm (C00-D49), n (%)	501 (27.8%)	11,517 (24.7%)	0.070	501 (27.8%)	474 (26.3%)	0.034
**Conditions not included in the propensity score model**						
Rheumatoid arthritis with rheumatoid factor (M05), n (%)	42 (2.2%)	170 (0.3%)	0.171	NR	NR	NR
Other rheumatoid arthritis (M06), n (%)	89 (4.8%)	780 (1.5%)	0.188	NR	NR	NR
Crohn disease (K50), n (%)	27 (1.4%)	295 (0.6%)	0.088	NR	NR	NR
Ulcerative colitis (K51), n (%)	37 (2.0%)	318 (0.6%)	0.121	NR	NR	NR
Psoriatic arthritis (L40.5), n (%)	42 (2.2%)	188 (0.4%)	0.167	NR	NR	NR
Enteropathic arthropathies (M07), n (%)	10 (0.5%)	18 (0.03%)	0.094	NR	NR	NR
Rheumatoid arthritis (M05–M06), n (%)	NR	NR	NR	97 (5.4%)	21 (1.2%)	0.239
Acne (L70), %	NR	NR	NR	19.3%	13.9%	0.146

Values are reported as mean ± SD or n (%). The original TriNetX query identified 1,871 JAKi-exposed and 51,930 unexposed patients; 1,799 and 48,367 patients, respectively, entered propensity score matching in the primary general safety analysis. Percentages are reproduced as reported by TriNetX. In the unmatched comparator cohort, 46,534 patients had evaluable age-at-index/demographic data; therefore, percentages

do not use the full PSM-pool denominator of 48,367. Only covariates displayed in the current primary TriNetX propensity score-matching output are included. Abbreviations: AA, alopecia areata; JAKi, Janus kinase inhibitor(s); PSM, propensity score matching; SD, standard deviation; SMD, standardized mean difference. Conditions in the lower panel were not included in the propensity score model. Before-matching values for these conditions refer to the full query cohorts (1,871 JAKi-exposed and 51,930 unexposed patients); after matching, the platform reported rheumatoid arthritis (M05–M06 combined) and acne for the primary general safety analysis (1,799 patients per cohort). NR, not reported.

Table S2. Primary pooled safety outcomes with nominal and Benjamini–Hochberg-adjusted p values and proportional hazards testing.

Outcome	Domain	Events / at risk (JAKi)	Events / at risk (no JAKi)	HR (95% CI)	Nominal p	BH-adjusted q	Significant after FDR	PH test p
Acne	General	145/1,451	83/1,549	2.425 (1.838–3.200)	<0.001	<0.001	Yes	0.632
Upper respiratory tract infections	General	84/1,432	199/1,428	0.620 (0.476–0.808)	<0.001	0.004	Yes	0.458
Urinary tract infections	General	25/1,704	91/1,672	0.458 (0.290–0.724)	0.001	0.006	Yes	0.037
Cutaneous cancer	Secondary	21/1,772	18/1,765	2.639 (1.322–5.270)	0.005	0.024	Yes	0.369
Substance use disorders	Psychiatric	30/1,639	95/1,640	0.602 (0.390–0.928)	0.020	0.070	No	0.010
Hyperlipidemia	Secondary	121/1,542	169/1,579	1.338 (1.043–1.716)	0.022	0.070	No	0.836
Venous thromboembolism	Cardiovascular	17/1,793	16/1,789	2.250 (1.062–4.769)	0.031	0.084	No	0.110
Suicide/self-harm	Psychiatric	12/1,742	12/1,757	2.166 (0.894–5.250)	0.081	0.192	No	0.272
Overall psychiatric composite	Psychiatric	107/1,241	224/1,226	0.812 (0.636–1.036)	0.093	0.196	No	0.010
Rheumatoid arthritis	Autoimmune	16/1,702	20/1,778	1.631 (0.802–3.317)	0.174	0.330	No	0.980
Herpes zoster	General	24/1,767	30/1,771	1.458 (0.826–2.576)	0.191	0.330	No	0.186
Psoriasis	Autoimmune	19/1,704	23/1,753	1.424 (0.749–2.710)	0.279	0.433	No	0.445
Overall general adverse events	General	187/1,111	251/1,163	1.111 (0.912–1.353)	0.296	0.433	No	0.944
Sjögren syndrome	Autoimmune	11/1,754	24/1,775	0.784 (0.373–1.646)	0.519	0.704	No	0.143
Composite autoimmune outcome	Autoimmune	52/1,400	104/1,574	0.918 (0.647–1.302)	0.630	0.798	No	0.036
Severe infections requiring hospitalization	General	31/1,746	61/1,712	1.101 (0.694–1.745)	0.683	0.811	No	0.760
Anxiety	Psychiatric	93/1,451	176/1,473	0.977 (0.748–1.277)	0.867	0.917	No	0.002
Autoimmune thyroiditis	Autoimmune	18/1,707	29/1,751	0.950 (0.513–1.759)	0.869	0.917	No	0.833
Depression	Psychiatric	62/1,527	116/1,544	1.007 (0.725–1.397)	0.968	0.968	No	0.102

Values are hazard ratios (HRs) with 95% confidence intervals (CIs). Nominal p values are from the primary pooled analysis. Benjamini–Hochberg adjustment was applied across the 19 estimable clinical outcomes in the pooled primary analysis to control the false discovery rate (FDR) at 5%; other analyses are reported with nominal p values. A BH-adjusted q value < 0.05 was considered statistically significant after multiplicity correction. PH test p values are from the TriNetX platform's built-in proportionality test; $p < 0.05$ indicates evidence against the proportional hazards assumption. Abbreviations: BH, Benjamini–Hochberg; CI, confidence interval; FDR, false discovery rate; HR, hazard ratio; JAKi, Janus kinase inhibitor(s); PH, proportional hazards.

Table S3. Sensitivity analyses varying the analytic window, with incident events and patients at risk.

Outcome	S1: 30-day post-index latency	S2: 5-year pre-index covariate window	S3: fixed 2-year follow-up	S5: 180-day post-index latency
**General safety outcomes**	*matched n = 1,811 per arm*	*matched n = 1,811 per arm*	*matched n = 2,161 per arm*	*matched n = 2,244 per arm*
Overall general adverse events	1.027 (0.844–1.249)<br>p = 0.793<br>*178/1,116 vs 270/1,183*	1.013 (0.837–1.226)<br>p = 0.893<br>*189/1,127 vs 293/1,190*	0.971 (0.789–1.196)*<br>p = 0.784<br>*166/1,334 vs 191/1,386*	0.962 (0.774–1.194)<br>p = 0.723<br>*136/1,312 vs 272/1,366*
Herpes zoster	1.703 (0.932–3.113)*<br>p = 0.080<br>*23/1,779 vs 24/1,778*	1.993 (1.083–3.667)*<br>p = 0.024<br>*24/1,780 vs 22/1,782*	1.288 (0.577–2.878)<br>p = 0.536<br>*13/2,118 vs 11/2,118*	1.515 (0.852–2.692)<br>p = 0.155<br>*22/2,196 vs 33/2,195*
Severe infections requiring hospitalization	0.877 (0.553–1.391)<br>p = 0.577<br>*29/1,757 vs 64/1,758*	1.082 (0.679–1.726)<br>p = 0.739<br>*31/1,759 vs 56/1,746*	0.757 (0.399–1.434)<br>p = 0.391<br>*16/2,083 vs 23/2,071*	1.088 (0.680–1.741)<br>p = 0.725<br>*29/2,153 vs 68/2,136*
Upper respiratory tract infections	0.648 (0.496–0.847)<br>p = 0.001<br>*82/1,439 vs 193/1,409*	0.556 (0.430–0.720)<br>p < 0.001<br>*85/1,442 vs 224/1,438*	0.558 (0.413–0.753)<br>p < 0.001<br>*66/1,703 vs 122/1,662*	0.652 (0.494–0.861)<br>p = 0.002<br>*74/1,737 vs 204/1,676*
Urinary tract infections	0.495 (0.316–0.776)<br>p = 0.002<br>*26/1,712 vs 90/1,688*	0.489 (0.312–0.767)<br>p = 0.001<br>*26/1,712 vs 91/1,673*	0.314 (0.176–0.558)<br>p < 0.001<br>*15/2,019 vs 51/1,987*	0.550 (0.354–0.855)<br>p = 0.007<br>*27/2,092 vs 108/2,039*
Acne	2.025 (1.551–2.643)<br>p < 0.001<br>*136/1,461 vs 102/1,579*	2.274 (1.740–2.972)<br>p < 0.001<br>*145/1,470 vs 96/1,595*	2.787 (2.068–3.755)<br>p < 0.001<br>*144/1,756 vs 62/1,912*	2.192 (1.609–2.987)<br>p < 0.001<br>*95/1,755 vs 81/1,930*
**Psychiatric outcomes**	*matched n = 2,244 per arm*	*matched n = 1,811 per arm*	*matched n = 2,160 per arm*	*matched n = 2,245 per arm*
Overall psychiatric composite	0.939 (0.745–1.184)<br>p = 0.596<br>*122/1,539 vs 249/1,565*	0.817 (0.645–1.034)*<br>p = 0.092<br>*114/1,272 vs 230/1,299*	0.792 (0.603–1.041)<br>p = 0.094<br>*90/1,469 vs 122/1,510*	0.923 (0.716–1.189)<br>p = 0.533<br>*97/1,514 vs 235/1,531*
Depression	1.074 (0.789–1.462)<br>p = 0.648<br>*69/1,918 vs 138/1,965*	0.902 (0.657–1.240)*<br>p = 0.525<br>*64/1,561 vs 119/1,601*	0.774 (0.529–1.132)<br>p = 0.186<br>*45/1,840 vs 65/1,892*	0.986 (0.703–1.383)<br>p = 0.935<br>*54/1,903 vs 134/1,973*
Anxiety	1.107 (0.854–1.434)<br>p = 0.444<br>*101/1,801 vs 191/1,858*	0.917 (0.706–1.192)*<br>p = 0.517<br>*95/1,484 vs 179/1,527*	0.914 (0.672–1.242)<br>p = 0.564<br>*74/1,733 vs 91/1,792*	1.116 (0.839–1.486)<br>p = 0.450<br>*79/1,779 vs 178/1,841*
Substance use disorders	0.780 (0.525–1.158)<br>p = 0.216<br>*37/2,071 vs 122/2,100*	0.624 (0.410–0.949)*<br>p = 0.026<br>*32/1,678 vs 97/1,696*	0.628 (0.371–1.062)<br>p = 0.080<br>*22/1,985 vs 38/2,010*	0.728 (0.473–1.119)<br>p = 0.146<br>*30/2,065 vs 106/2,096*
**Autoimmune outcomes**	*matched n = 1,811 per arm*	*matched n = 1,811 per arm*	*matched n = 2,161 per arm*	*matched n = 2,172 per arm*
Composite autoimmune outcome	0.816 (0.577–1.154)*<br>p = 0.248<br>*50/1,395 vs 112/1,554*	0.894 (0.633–1.263)*<br>p = 0.526<br>*53/1,398 vs 104/1,572*	0.838 (0.571–1.229)<br>p = 0.365<br>*45/1,657 vs 63/1,820*	1.020 (0.697–1.491)<br>p = 0.920<br>*43/1,646 vs 94/1,810*
Rheumatoid arthritis	1.559 (0.780–3.116)<br>p = 0.206<br>*16/1,712 vs 21/1,786*	2.086 (0.998–4.360)<br>p = 0.047<br>*16/1,712 vs 17/1,789*	1.114 (0.516–2.405)<br>p = 0.783<br>*13/2,032 vs 13/2,129*	*< 10 events*<br>*estimate suppressed*

Outcome	S1: 30-day post-index latency	S2: 5-year pre-index covariate window	S3: fixed 2-year follow-up	S5: 180-day post-index latency
Psoriasis	0.979 (0.545–1.759)<br>p = 0.943<br>*19/1,711 vs 36/1,763*	1.085 (0.601–1.959)<br>p = 0.786<br>*20/1,712 vs 31/1,753*	0.748 (0.395–1.416)<br>p = 0.370<br>*16/2,047 vs 23/2,081*	1.350 (0.711–2.562)<br>p = 0.358<br>*17/2,048 vs 31/2,094*
Autoimmune thyroiditis	0.902 (0.454–1.789)<br>p = 0.767<br>*14/1,713 vs 24/1,755*	1.220 (0.625–2.381)<br>p = 0.560<br>*17/1,716 vs 21/1,770*	1.083 (0.553–2.123)<br>p = 0.816<br>*17/2,046 vs 17/2,098*	0.698 (0.339–1.438)<br>p = 0.327<br>*11/2,044 vs 28/2,093*
**Other outcomes**	*matched n = 1,811 per arm*	*matched n = 1,811 per arm*	*matched n = 2,160 per arm*	*matched n = 2,172 per arm*
Venous thromboembolism	2.506 (1.138–5.519)<br>p = 0.019<br>*16/1,800 vs 14/1,794*	1.896 (0.896–4.008)*<br>p = 0.090<br>*16/1,800 vs 17/1,796*	*< 10 events*<br>*estimate suppressed*	*< 10 events*<br>*estimate suppressed*
Hyperlipidemia	1.306 (1.015–1.682)<br>p = 0.038<br>*116/1,535 vs 171/1,568*	1.168 (0.919–1.485)<br>p = 0.204<br>*124/1,543 vs 190/1,568*	1.419 (1.066–1.890)<br>p = 0.016<br>*108/1,813 vs 83/1,842*	1.411 (1.101–1.808)<br>p = 0.006<br>*119/1,779 vs 194/1,785*
Cutaneous cancer	2.554 (1.224–5.327)<br>p = 0.010<br>*18/1,782 vs 16/1,775*	2.299 (1.142–4.627)<br>p = 0.017<br>*19/1,783 vs 17/1,780*	1.636 (0.759–3.527)<br>p = 0.205<br>*16/2,118 vs 11/2,122*	2.727 (1.440–5.164)<br>p = 0.001<br>*25/2,129 vs 19/2,124*
Negative control outcome: injuries to the shoulder and upper arm	1.045 (0.641–1.702)*<br>p = 0.860<br>*28/1,733 vs 54/1,721*	0.770 (0.489–1.212)<br>p = 0.257<br>*29/1,734 vs 73/1,740*	0.637 (0.356–1.139)<br>p = 0.125<br>*18/2,055 vs 31/2,054*	0.886 (0.561–1.400)<br>p = 0.604<br>*29/2,060 vs 73/2,037*

All four analyses used the primary cohorts and the primary covariate set; each varied one analytic parameter. S1 began the outcome window 30 days after the index event and S5 180 days after it; S2 ascertained the propensity score covariates within the 1825 days preceding the index event rather than over the entire available record; S3 restricted follow-up to days 1–730 after index. S1 and S2 were run on 19 May 2026, S3 on 22 August 2026, S5 and the psychiatric domain of S1 on 26 September 2026.

Values are hazard ratios (HRs) with 95% confidence intervals (CIs) from Cox proportional hazards models; p values are nominal and derived from log-rank tests. Italicised figures below each estimate are incident events / patients at risk in the JAKi cohort versus the comparator cohort, after exclusion of patients with the outcome before the start of the time window.

Propensity score matching was performed separately for each analysis and outcome domain, and the network was re-queried at each run; the matched cohort size per arm is therefore given for every analysis and domain.

* Evidence against the proportional hazards assumption (PH test $p < 0.05$); the corresponding HR should be interpreted as a summary estimate over follow-up rather than as a constant relative hazard.

"< 10 events" indicates that the outcome was analysed but the patient count was below the reporting threshold of the TriNetX platform, which then returns "Based on the outcome terms, the patient count is too small, so detailed results cannot be displayed" and releases no counts or estimate. These cells denote absence of information, not absence of effect.

Abbreviations: AA, alopecia areata; CI, confidence interval; HR, hazard ratio; JAKi, Janus kinase inhibitor(s); PH, proportional hazards.

Table S4. Sensitivity analyses varying the study population or the comparator, with incident events and patients at risk.

Outcome	S4: active comparator	S6: index on/after 1 June 2022	S7: ≥1 medication event	S8: JAKi-indication diseases excluded
**General safety outcomes**	*matched n = 1,805 per arm*	*matched n = 1,759 per arm*	*matched n = 3,249 per arm*	*matched n = 1,939 per arm*
Overall general adverse events	0.896 (0.737–1.090)<br>p = 0.272<br>*175/1,079 vs 257/1,061*	1.149 (0.907–1.455)<br>p = 0.249<br>*155/1,084 vs 124/1,050*	0.978 (0.844–1.134)<br>p = 0.771<br>*314/2,068 vs 475/2,075*	1.070 (0.881–1.299)<br>p = 0.494<br>*191/1,248 vs 281/1,239*
Herpes zoster	0.726 (0.428–1.229)<br>p = 0.231<br>*21/1,765 vs 49/1,750*	*< 10 events*<br>*estimate suppressed*	1.524 (0.959–2.422)<br>p = 0.073<br>*35/3,188 vs 45/3,190*	0.851 (0.461–1.572)<br>p = 0.607<br>*17/1,908 vs 33/1,910*
Severe infections requiring hospitalization	0.455 (0.293–0.705)*<br>p < 0.001<br>*27/1,731 vs 95/1,663*	0.562 (0.302–1.048)<br>p = 0.066<br>*16/1,700 vs 26/1,691*	0.963 (0.687–1.349)<br>p = 0.825<br>*56/3,127 vs 114/3,110*	0.691 (0.389–1.229)<br>p = 0.206<br>*17/1,878 vs 56/1,872*
Upper respiratory tract infections	0.551 (0.420–0.723)<br>p < 0.001<br>*76/1,401 vs 193/1,334*	0.497 (0.357–0.693)<br>p < 0.001<br>*56/1,393 vs 92/1,285*	0.605 (0.498–0.734)<br>p < 0.001<br>*154/2,574 vs 355/2,479*	0.608 (0.461–0.800)<br>p < 0.001<br>*76/1,571 vs 210/1,482*
Urinary tract infections	0.379 (0.240–0.596)*<br>p < 0.001<br>*24/1,672 vs 104/1,618*	0.372 (0.200–0.694)<br>p = 0.001<br>*14/1,651 vs 34/1,637*	0.500 (0.357–0.701)<br>p < 0.001<br>*46/3,016 vs 158/2,978*	0.580 (0.346–0.972)<br>p = 0.037<br>*21/1,843 vs 63/1,786*
Acne	1.688 (1.321–2.157)<br>p < 0.001<br>*143/1,455 vs 128/1,577*	2.199 (1.638–2.952)<br>p < 0.001<br>*136/1,417 vs 66/1,503*	1.848 (1.516–2.253)<br>p < 0.001<br>*231/2,725 vs 191/2,880*	2.251 (1.753–2.890)<br>p < 0.001<br>*166/1,590 vs 114/1,676*
**Psychiatric outcomes**	*matched n = 1,796 per arm*	*matched n = 1,759 per arm*	*matched n = 3,242 per arm*	*matched n = 1,939 per arm*
Overall psychiatric composite	0.878 (0.683–1.130)<br>p = 0.312<br>*101/1,188 vs 196/1,175*	0.683 (0.502–0.928)<br>p = 0.014<br>*74/1,201 vs 91/1,156*	0.979 (0.820–1.169)<br>p = 0.813<br>*213/2,228 vs 367/2,286*	0.687 (0.542–0.871)<br>p = 0.002<br>*105/1,391 vs 258/1,384*
Depression	0.903 (0.656–1.244)<br>p = 0.533<br>*61/1,510 vs 126/1,517*	0.684 (0.454–1.029)<br>p = 0.067<br>*40/1,500 vs 54/1,527*	1.011 (0.799–1.280)<br>p = 0.924<br>*120/2,778 vs 211/2,837*	0.855 (0.617–1.185)<br>p = 0.346<br>*59/1,701 vs 132/1,717*
Anxiety	1.121 (0.850–1.480)<br>p = 0.418<br>*88/1,429 vs 155/1,467*	0.722 (0.519–1.005)<br>p = 0.053<br>*64/1,406 vs 78/1,378*	1.063 (0.873–1.294)<br>p = 0.543<br>*173/2,610 vs 291/2,707*	0.827 (0.629–1.088)<br>p = 0.174<br>*83/1,599 vs 186/1,613*
Substance use disorders	0.853 (0.548–1.328)<br>p = 0.481<br>*31/1,635 vs 71/1,643*	0.498 (0.271–0.913)*<br>p = 0.021<br>*16/1,614 vs 30/1,632*	0.799 (0.594–1.075)<br>p = 0.138<br>*69/2,999 vs 154/3,024*	0.720 (0.457–1.133)*<br>p = 0.154<br>*28/1,814 vs 87/1,840*
**Autoimmune outcomes**	*matched n = 1,805 per arm*	*matched n = 1,759 per arm*	*matched n = 3,249 per arm*	*matched n = 1,939 per arm*
Composite autoimmune outcome	0.494 (0.350–0.698)<br>p < 0.001<br>*51/1,371 vs 106/972*	0.879 (0.565–1.367)<br>p = 0.566<br>*37/1,379 vs 42/1,471*	1.012 (0.787–1.302)<br>p = 0.924<br>*103/2,509 vs 180/2,787*	*excluded by design*

Outcome	S4: active comparator	S6: index on/after 1 June 2022	S7: ≥1 medication event	S8: JAKi-indication diseases excluded
Rheumatoid arthritis	0.404 (0.233–0.699)<br>p = 0.001<br>*17/1,692 vs 65/1,552*	*< 10 events*<br>*estimate suppressed*	1.334 (0.745–2.388)<br>p = 0.330<br>*22/3,069 vs 30/3,200*	*excluded by design*
Psoriasis	0.608 (0.350–1.057)<br>p = 0.075<br>*19/1,707 vs 43/1,602*	0.944 (0.438–2.036)<br>p = 0.883<br>*13/1,666 vs 13/1,700*	1.315 (0.880–1.966)*<br>p = 0.180<br>*47/3,083 vs 58/3,137*	0.771 (0.389–1.529)<br>p = 0.456<br>*13/1,880 vs 30/1,896*
Autoimmune thyroiditis	0.752 (0.392–1.445)<br>p = 0.391<br>*14/1,709 vs 29/1,727*	*< 10 events*<br>*estimate suppressed*	1.062 (0.655–1.721)<br>p = 0.808<br>*30/3,083 vs 44/3,161*	0.986 (0.525–1.851)<br>p = 0.965<br>*17/1,845 vs 30/1,879*
**Other outcomes**	*matched n = 1,796 per arm*	*matched n = 1,759 per arm*	*matched n = 3,249 per arm*	*matched n = 1,939 per arm*
Venous thromboembolism	*< 10 events*<br>*estimate suppressed*	*< 10 events*<br>*estimate suppressed*	1.393 (0.792–2.452)<br>p = 0.248<br>*24/3,221 vs 29/3,229*	*< 10 events*<br>*estimate suppressed*
Hyperlipidemia	1.591 (1.246–2.033)<br>p < 0.001<br>*135/1,478 vs 155/1,456*	1.055 (0.813–1.370)<br>p = 0.687<br>*122/1,478 vs 105/1,476*	1.238 (1.040–1.474)<br>p = 0.016<br>*237/2,719 vs 359/2,775*	1.214 (0.966–1.524)<br>p = 0.095<br>*138/1,675 vs 222/1,723*
Cutaneous cancer	1.960 (1.089–3.529)<br>p = 0.023<br>*25/1,758 vs 26/1,753*	*< 10 events*<br>*estimate suppressed*	1.627 (1.037–2.553)*<br>p = 0.033<br>*39/3,183 vs 53/3,206*	1.680 (0.859–3.289)*<br>p = 0.126<br>*19/1,907 vs 20/1,906*
Negative control outcome: injuries to the shoulder and upper arm	0.817 (0.511–1.309)<br>p = 0.401<br>*27/1,709 vs 65/1,690*	0.622 (0.336–1.152)<br>p = 0.128<br>*17/1,678 vs 25/1,649*	0.788 (0.557–1.113)<br>p = 0.175<br>*50/3,098 vs 117/3,100*	0.649 (0.400–1.052)<br>p = 0.077<br>*24/1,860 vs 78/1,865*

S4 replaced the comparator with patients receiving conventional systemic immunosuppressants (sulfasalazine, mycophenolic acid/mycophenolate mofetil, azathioprine, oral cyclosporine, or methotrexate); autoimmune outcomes were not interpreted in S4 because conventional immunosuppressant use may itself reflect autoimmune disease. S6 required all cohort-qualifying terms to have occurred on or after 1 June 2022, the month in which the first JAK inhibitor was approved for alopecia areata (baricitinib; US Food and Drug Administration, 13 June 2022), so that both cohorts were drawn from the same calendar period; mean follow-up was then 610 days in the JAKi cohort and 567 days in the comparator, against 851 and 1,638 days in the primary analysis. S7 required one rather than two JAK inhibitor medication events, with the index date set at the first medication event, removing the requirement that patients persist to a second dispensing. S8 excluded from both cohorts any patient with a recorded diagnosis of rheumatoid arthritis (M05, M06), inflammatory bowel disease (K50, K51), psoriatic or enteropathic arthropathy (L40.5, M07) or ankylosing spondylitis (M45), and added glucocorticoids (VA class HS051) to the propensity score covariates. S4 was run on 22 August 2026, S6 to S8 on 27 September 2026.

"Excluded by design" marks outcomes that cannot be estimated in S8 because the defining diagnoses were removed from both cohorts; the composite autoimmune outcome is not reported in S8 for the same reason, since six of its component codes were excluded.

In S8, glucocorticoid exposure (VA class HS051) was recorded in 69.6% of the JAKi cohort and 50.4% of the comparator before matching (standardized mean difference 0.400) and in 69.6% and 67.7% after matching (standardized mean difference 0.041).

Values are hazard ratios (HRs) with 95% confidence intervals (CIs) from Cox proportional hazards models; p values are nominal and derived from log-rank tests. Italicised figures below each estimate are incident events / patients at risk in the JAKi cohort versus the comparator cohort, after exclusion of patients with the outcome before the start of the time window.

Propensity score matching was performed separately for each analysis and outcome domain, and the network was re-queried at each run; the matched cohort size per arm is therefore given for every analysis and domain.

* Evidence against the proportional hazards assumption (PH test $p < 0.05$); the corresponding HR should be interpreted as a summary estimate over follow-up rather than as a constant relative hazard.

"< 10 events" indicates that the outcome was analysed but the patient count was below the reporting threshold of the TriNetX platform, which then returns "Based on the outcome terms, the patient count is too small, so detailed results cannot be displayed" and releases no counts or estimate. These cells denote absence of information, not absence of effect.

Abbreviations: AA, alopecia areata; CI, confidence interval; HR, hazard ratio; JAKi, Janus kinase inhibitor(s); PH, proportional hazards.

Table S5. Approximate incidence rates per 100 person-years in the fixed 2-year follow-up analysis (S3).

Outcome	JAKi events	JAKi person-years	JAKi rate per 100 PY (95% CI)	Comparator events	Comparator person-years	Comparator rate per 100 PY (95% CI)	Incidence rate ratio (95% CI)
Overall general adverse events	166	1,858	8.93 (7.63–10.40)	191	2,117	9.02 (7.79–10.40)	0.99 (0.80–1.22)
Herpes zoster	13	2,950	0.44 (0.23–0.75)	11	3,235	0.34 (0.17–0.61)	1.30 (0.58–2.89)
Severe infections requiring hospitalization	16	2,901	0.55 (0.32–0.90)	23	3,163	0.73 (0.46–1.09)	0.76 (0.40–1.44)
Upper respiratory tract infections	66	2,372	2.78 (2.15–3.54)	122	2,538	4.81 (3.99–5.74)	0.58 (0.43–0.78)
Urinary tract infections	15	2,812	0.53 (0.30–0.88)	51	3,034	1.68 (1.25–2.21)	0.32 (0.18–0.56)
Acne	144	2,446	5.89 (4.97–6.93)	62	2,920	2.12 (1.63–2.72)	2.77 (2.06–3.73)
Overall psychiatric composite	90	2,046	4.40 (3.54–5.41)	122	2,306	5.29 (4.39–6.32)	0.83 (0.63–1.09)
Depression	45	2,563	1.76 (1.28–2.35)	65	2,889	2.25 (1.74–2.87)	0.78 (0.53–1.14)
Anxiety	74	2,414	3.07 (2.41–3.85)	91	2,737	3.33 (2.68–4.08)	0.92 (0.68–1.25)
Substance use disorders	22	2,765	0.80 (0.50–1.20)	38	3,070	1.24 (0.88–1.70)	0.64 (0.38–1.09)
Composite autoimmune outcome	45	2,308	1.95 (1.42–2.61)	63	2,779	2.27 (1.74–2.90)	0.86 (0.59–1.26)
Rheumatoid arthritis	13	2,830	0.46 (0.24–0.79)	13	3,251	0.40 (0.21–0.68)	1.15 (0.53–2.48)
Psoriasis	16	2,851	0.56 (0.32–0.91)	23	3,178	0.72 (0.46–1.09)	0.78 (0.41–1.47)
Autoimmune thyroiditis	17	2,850	0.60 (0.35–0.96)	17	3,204	0.53 (0.31–0.85)	1.12 (0.57–2.20)
Hyperlipidemia	108	2,525	4.28 (3.51–5.16)	83	2,813	2.95 (2.35–3.66)	1.45 (1.09–1.93)
Cutaneous cancer	16	2,950	0.54 (0.31–0.88)	11	3,241	0.34 (0.17–0.61)	1.60 (0.74–3.44)
Negative control: injuries to the shoulder and upper arm	18	2,862	0.63 (0.37–0.99)	31	3,137	0.99 (0.67–1.40)	0.64 (0.36–1.14)

Person-years were approximated as the number of patients at risk multiplied by the cohort mean follow-up (508.7 days in the JAKi cohort and 557.8 days in the comparator cohort), because the TriNetX platform does not report outcome-specific person-time; rates should therefore be interpreted as approximations. Patients at risk exclude those with the outcome before the start of the time window. Confidence intervals for rates are exact Poisson intervals; confidence intervals for incidence rate ratios were calculated on the log scale.

Abbreviations: CI, confidence interval; JAKi, Janus kinase inhibitor(s); PY, person-years.

Table S6. Analysis run dates and cohort sizes.

Analysis	Run date	Matched patients per arm (range across outcome domains)
Primary analysis	2 May 2026	1,771–1,805
S1: 30-day post-index latency	19 May 2026 (psychiatric domain: 26 September 2026)	1,811 (psychiatric domain: 2,244)
S2: 5-year pre-index covariate window	19 May 2026	1,811
S3: fixed 2-year follow-up	22 August 2026	2,160–2,161
S4: active comparator	22 August 2026	1,796–1,805
S5: 180-day post-index latency	26 September 2026	2,172–2,245
S6: index on/after 1 June 2022	27 September 2026	1,759
S7: ≥1 medication event	27 September 2026	3,242–3,249
S8: JAKi-indication diseases excluded	27 September 2026	1,939
Drug-specific: baricitinib	26 September 2026	1,241–1,242
Drug-specific: tofacitinib	26 September 2026	778
Drug-specific: ritlecitinib	26 September 2026	485
Drug-specific: oral ruxolitinib	26 September 2026	42

The TriNetX platform reports, on the query definition page of each report, the cohort size stored at the last execution of the cohort query (1,871 JAKi-exposed and 51,930 unexposed patients for the primary cohort definitions). Because the network is continuously updated and cohorts are re-queried at each run, cohort sizes at later runs can exceed the stored query counts; for example, 2,161 JAKi-exposed patients were available in S3 on 22 August 2026, more than the 1,871 patients stored for the query.

Dermatologic negative control outcomes, analysed on 26 September 2026 (incident events / patients at risk, JAKi vs. comparator): melanocytic nevi (D22), HR 1.197 (95% CI, 0.893–1.604), p = 0.229, 82/1,924 vs. 132/1,910; seborrheic keratosis (L82), HR 1.279 (95% CI, 0.950–1.724), p = 0.104, 83/1,980 vs. 113/1,965; encounter for screening for malignant neoplasm of skin (Z12.83), HR 1.153 (95% CI, 0.695–1.912), p = 0.580, 26/2,125 vs. 51/2,090. There was no evidence against the proportional hazards assumption for any of these outcomes (PH test p = 0.256, 0.830, and 0.546, respectively).

Abbreviations: JAKi, Janus kinase inhibitor(s).

Figure S1. Love plot showing baseline covariate balance before and after propensity score matching in patients with alopecia areata treated with JAK inhibitors versus non-users.

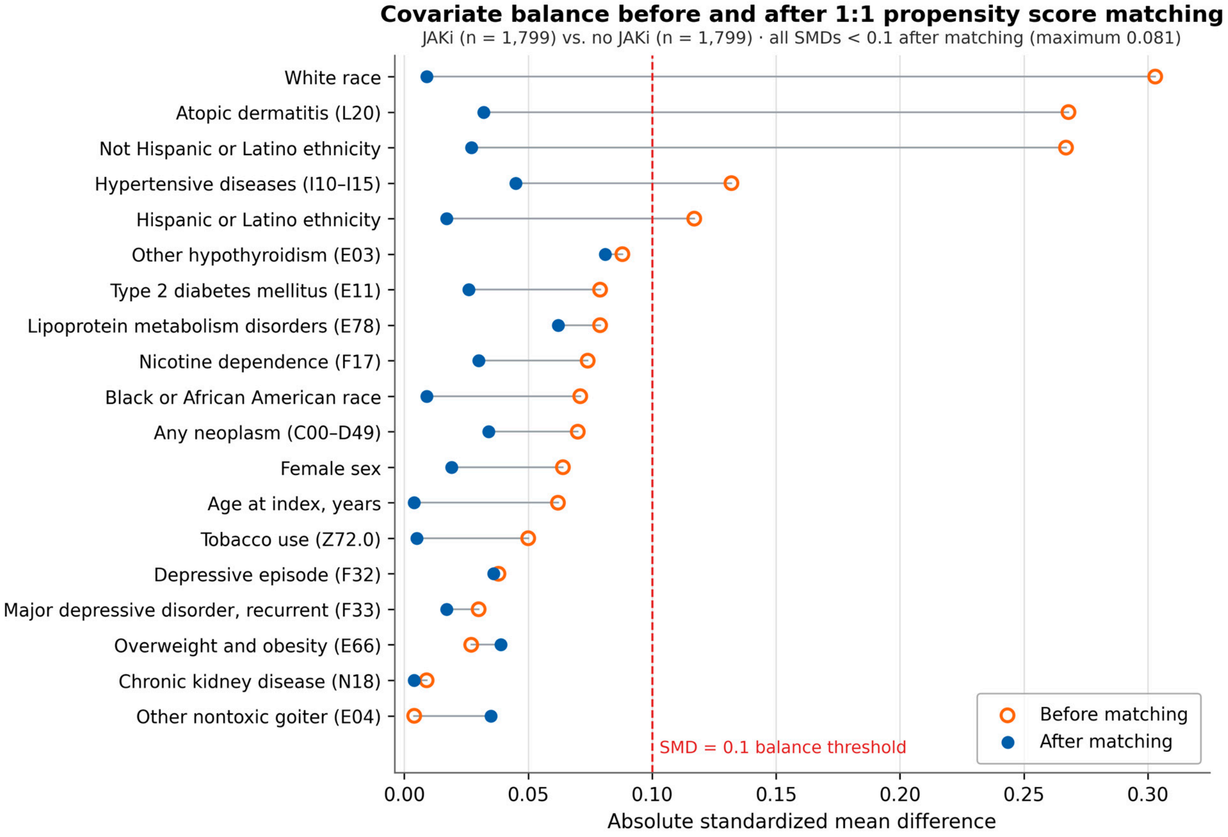

Each marker represents the absolute standardized mean difference (SMD) for one covariate; the dashed line marks the prespecified balance threshold of SMD = 0.1. All covariates were balanced after matching (maximum SMD 0.081). Abbreviations: JAKi, Janus kinase inhibitor(s); SMD, standardized mean difference.
